# A Large-Scale Study of Anxiety and Depression in People with Multiple Sclerosis: A Survey via the Web Portal of the UK MS Register

**DOI:** 10.1371/journal.pone.0041910

**Published:** 2012-07-30

**Authors:** Kerina H. Jones, David V. Ford, Philip A. Jones, Ann John, Rodden M. Middleton, Hazel Lockhart-Jones, Lisa A. Osborne, J. Gareth Noble

**Affiliations:** 1 College of Medicine, Swansea University, Swansea, Wales, United Kingdom; 2 Long Term and Chronic Conditions Centre, College of Human and Health Sciences, Swansea University, Swansea, Wales, United Kingdom; Innsbruck Medical University, Austria

## Abstract

**Introduction:**

Studies have found that people with Multiple Sclerosis experience relatively high rates of anxiety and depression. Although methodologically robust, many of these studies had access to only modest sample sizes (N<200). The aims of this study were to use responses gained via the web portal of the UK MS Register (N>4000) to: describe the depression and anxiety profiles of people with MS; to determine if anxiety and depression are related to age or disease duration; and to assess whether the levels of anxiety and depression differ between genders and types of MS.

**Methods:**

From its launch in May 2011 to the end of December 2011, 7786 adults with MS enrolled to take part in the UK MS Register via the web portal. The responses to the Hospital Anxiety and Depression Scale (HADS) were collated with basic demographic and descriptive MS data provided at registration and the resulting dataset was analysed in SPSS (v.16).

**Results:**

The mean HADS score among the 4178 respondents was 15.7 (SE 0.117, SD 7.55) with a median of 15.0 (IQR 11). Anxiety and depression rates were notably high, with over half (54.1%) scoring ≥8 for anxiety and 46.9% scoring ≥8 for depression. Women with relapsing-remitting MS were more anxious than men with this type (*p*<0.001), and than women with other types of MS (*p* = 0.017). Within each gender, men and women with secondary progressive MS were more depressed than men or women with other types of MS (*p*<0.001, *p*<0.001).

**Conclusions:**

This largest known study of its kind has shown that anxiety and depression are highly prevalent in people with MS, indicating that their mental health needs could be better addressed. These findings support service planning and further research to provide the best care for people with MS to help alleviate these debilitating conditions.

## Introduction

Multiple Sclerosis (MS) is a chronic, inflammatory, autoimmune disease. The lifetime risk of major depression in people with MS has been estimated to be as high as 50% compared to 10 to 15% in the general population [1], [2] Also, studies have variously reported significantly higher rates of anxiety and depression in people with MS compared to the general population [1]–[4]. However, although methodologically robust, many of these studies used fairly modest sample sizes (N<200), with far fewer in excess of 400 [1], [2] . This was often because they relied on patients attending a clinic, or because the studies included other number-limiting factors such as neuropsychiatric and clinical examinations, or they were part of a clinical trial [1], [2], [5]–[7]. Because of the relatively high prevalence of anxiety and depression and the debilitation they can cause, we sought to add to the knowledge base on these conditions in people with MS using over 4,000 responses to the Hospital Anxiety and Depression Scale (HADS), gained via the web portal of the UK MS Register.

The UK MS Register captures data from three main sources and is able to anonymously link these data at the individual level whilst retaining privacy. Data are collected directly from people with MS via a purpose-built web portal, from sources of routine administrative data, and from patient-management systems operating in NHS neurology clinics. The web portal has been collecting data since it was launched in May 2011, and data collection from clinics and administrative sources is underway. By uniting data from three disparate sources the UK MS Register model is innovative in its design and provides new opportunities for studying MS via linked data. The Register is based on the proven technologies and robust Information Governance arrangements in place in the Secure Anonymised Information Linkage (SAIL) system developed by the Health Information Research Unit (HIRU) [8], [9].

The web portal functions as a questionnaire delivery platform for people with MS to provide information on their experiences of living with MS. It hosts a number of validated questionnaires, including: the Hospital Anxiety and Depression Scale [10], the MS Disease Impact Scale–29 [11], and the EQ-5D [12]. These cover a range of topics such as: MS and mental well-being; impact of MS on daily life; lifestyle and health outcomes. Baseline information including: age, gender, date of diagnosis of MS, type of MS and age at onset is collected as part of the registration process. We have shown that it is feasible to collect information in this way and to use it to characterize a cohort of people with MS [13].

### Research Aims

This is a novel study as it utilized responses received via the web portal of the UK MS Register to: profile anxiety and depression in people with MS; to explore the relationships between anxiety and depression and factors such as age and disease duration; and to assess whether the levels of anxiety and depression differ between genders and types of MS. With the relatively large sample size, we were able to make a reasonably in-depth address of these research aims, and it is anticipated that the findings will be useful to inform the mental health care of people with MS and to support further research.

## Methods

### Research Ethics and Governance

The UK MS Register study was peer-reviewed via the MS Society and it received ethical approval from the South West – Central Bristol Research Ethics Committee (11/SW/0160) as a research database [14]. Under this ethical approval, data collected via the portal, the neurology clinics and routine administrative sources can be anonymously linked using the SAIL methodologies provided that agreement to the portal terms of service (via the portal) and written informed participant consent (at the clinics) have been obtained. The working UK MS Register contains only anonymous data but with facilities are in place to re-contact participants to take part in further research [15]. In future, the Register data will be made accessible for analysis by researchers external to the team, subject to regulatory and governance requirements. The final operating model for these arrangements is yet to be determined, but we are able at accommodate researcher requests to view the data in the interim, subject to any necessary amendments to regulatory and governance approvals and a non-disclosure agreement.

### Data Collection and Analysis

Adults with MS living in the UK have been able to enroll on the web portal of the UK MS Register since its launch in May 2011. By the end of 2011, 7786 people had registered and data collection is on-going. The anxiety and depression components of the HADS are each robust, and the scale has been validated for use with people who have MS [10], [16]. The responses to the HADS received between May and December 2011 were collated with basic demographic and descriptive MS data provided at registration and the resulting dataset was analysed in SPSS (v.16). The findings were compared with normative data from a large non-clinical sample as a reference group [17]. Descriptive statistics were used to characterize the cohort of people with MS with respect to their anxiety and depression profiles. The continuous variables were assessed for normality using the Kolmogorov-Smirnov test and all were found to deviate significantly from the normal distribution (*p*<0.001). Because of this, non-parametric inferential tests were used: Spearman’s rank correlation coefficient to measure relationships between variables, the Chi square test was used to assess independence between categories, the Mann-Whitney U test was used to assess differences between two independent samples, and the Kruskal-Wallis one-way analysis of variance was used to compare more than two independent samples. Significance values are reported in the results and mean ranks are tabulated in a supplementary file (Table S1). A Bonferrioni correction was used where relevant to avoid over-reporting significance where multiple tests were conducted. This was calculated as the significance level divided by the number of tests undertaken.

**Table 1:** Timelines and HADS score of people with MS.

	N	Mean	SD	SE	Median	IQR	Range
**Age (yrs):**							
All	4617	50.9	11.5	0.17	51.0	16	20 to 87
Male	1355	52.8	11.4	0.31	53.0	17	23 to 87
Female	3253	50.5	11.4	0.20	50.0	16	20 to 84
**Time from first symptoms (yrs):**							
All	3765	16.7	11.3	0.18	15.0	16	0 to 63
Male	1065	16.9	11.4	0.35	15.0	16	0 to 62
Female	2627	16.6	11.3	0.22	15.0	16	0 to 63
**Time since diagnosis (yrs):**							
All	2265	12.2	9.4	0.20	10.0	13	0 to 47
Male	655	12.8	9.5	0.37	11.0	14	0 to 47
Female	1566	12.0	9.4	0.24	10.0	13	0 to 46
**HADS score:**							
All	4178	15.7	7.5	0.12	15.0	11	0 to 40
Male	1187	15.6	7.6	0.22	15.0	11	0 to 40
Female	2903	15.7	7.5	0.14	15.0	11	0 to 40
**Anxiety score:**							
All	4381	8.2	4.3	0.07	8.0	6	0 to 21
Male	1247	7.6	4.3	0.12	7.0	7	0 to 21
Female	3040	8.4	4.2	0.08	8.0	6	0 to 21
**Depression score:**							
All	4379	7.6	4.2	0.06	7.0	7	0 to 21
Male	1258	8.0	4.3	0.12	8.0	6	0 to 20
Female	3032	7.3	4.2	0.08	7.0	6	0 to 21

Descriptions of the variables are shown. Slight differences in totals by gender compared to all are due the small percentages (<2%) of participants for whom either age or gender was missing.

## Results

### Description of Respondents

**Figure 1:**
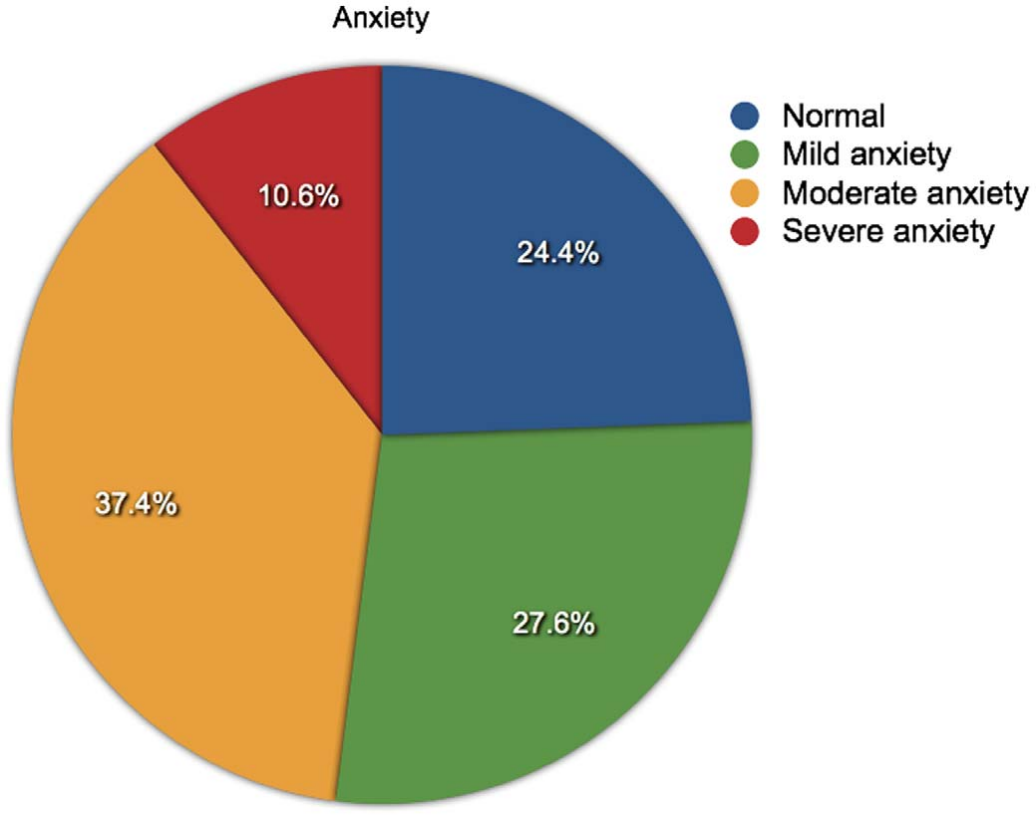
Levels of anxiety in people with a depression score > = 8. The proportions of people with different levels of anxiety (normal, mild, moderate or severe) and who have a depression score of 8 or above (N = 1961).

**Figure 2:**
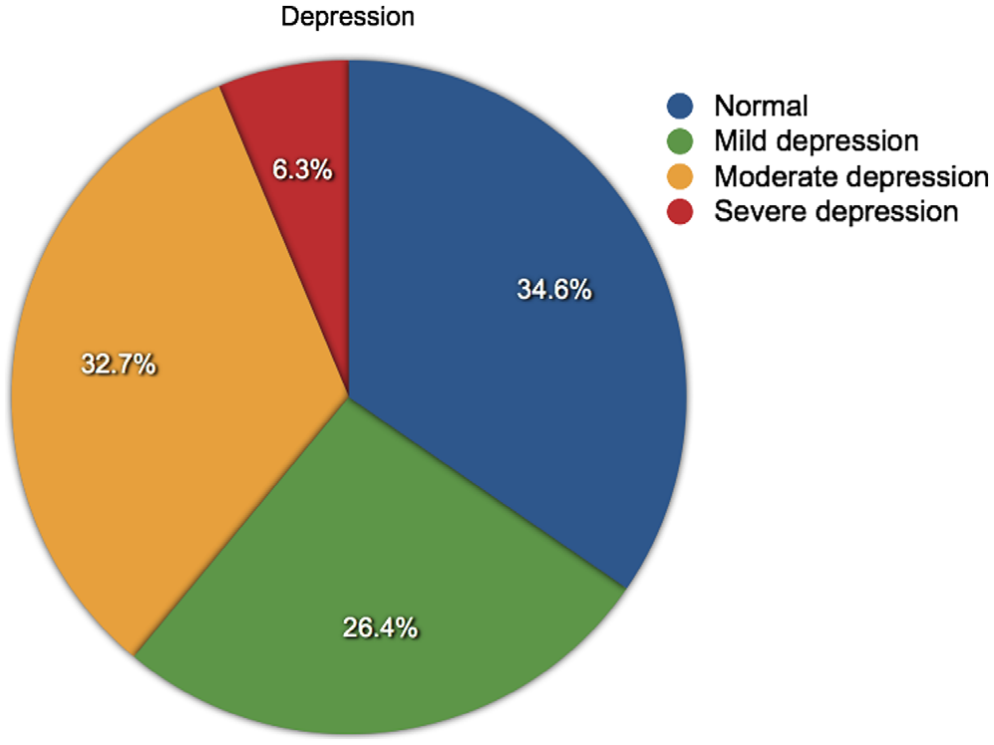
Levels of depression in people with an anxiety score > = 8. The proportions of people with different levels of depression (normal, mild, moderate or severe) and who have an anxiety score of 8 or above (N = 2268).

Among the respondents, 70.4% were women, 29.3% were men (2.4 women: 1 man) and 0.3% did not record their gender (*N* = 4706). The reported types of MS were: 14.4% primary progressive (PPMS), 61.7% relapsing-remitting (RRMS), 9.4% secondary progressive (SPMS), and 14.5% didn’t know their type of MS (DKMS) (*N* = 4540). The timelines and HADS scores of the respondents were explored using descriptive statistics. The mean age of the respondents was 50.9 years (SE 0.17, SD 11.5) with a median of 51.0 years (IQR 16). The mean time from first experiencing symptoms (disease onset) was 16.7 years (SE 0.16, SD 11.3) with a median of 15.0 years (IQR 16). The mean time since diagnosis (by a neurologist) was 12.2 years (SE 0.20, SD 9.4) with a median of 10.0 years (IQR 13). The mean HADS score was 15.7 (SE 0.12, SD 7.5) with a median of 15.0 (IQR 11). The mean anxiety score was 8.2 (SE 0.07, SD 4.3) with a median of 8.0 (IQR 6). The mean depression score was 7.6 (SE 0.06, SD 4.2) with a median of 7.0 (IQR 7). In the UK population reference group the mean values for anxiety and depression were 6.14 (SD 3.8, median 6) and 3.68 (SD 3.1, median 3), respectively [17]. The timelines and HADS scores are shown in more detail in Table 1, including the values by gender.

**Table 2:** Anxiety and depression levels in people with MS.

Categories	Anxiety and Depression				
	Normal	Mild	Moderate	Severe	Total
	< = 7	8–11	12–15	>15	
	Anxiety levels				
**Gender**					
Male: No.	649	277	254	67	1247
**%**	52.0	22.2	20.4	5.4	100.0
Female: No.	1319	801	749	171	3040
**%**	43.4	26.3	24.6	5.6	100.0
	**Depression levels**				
**Gender:**					
Male: No.	621	285	284	68	1258
**%**	49.4	22.7	22.6	5.4	100.0
Female: No.	1658	655	621	98	3032
**%**	54.7	21.6	20.5	3.2	100.0
	**Anxiety levels**				
**MS type**					
Primary progressive: No.	321	144	109	33	607
**%**	52.9	23.7	18.0	5.4	100.0
Relapsing-remitting: No.	1137	676	670	132	2615
**%**	43.5	25.9	25.6	5.0	100.0
Secondaryprogressive: No.	192	97	83	26	398
**%**	48.2	24.4	20.9	6.5	100.0
Don’t know: No.	290	147	131	45	613
**%**	47.3	24.0	21.4	7.3	100.0
	**Depression levels**				
**MS type:**					
Primary progressive: No.	314	145	124	29	612
**%**	51.3	23.7	20.3	4.7	100.0
Relapsing-remitting: No.	1,443	557	521	80	2601
**%**	55.5	21.4	20.0	3.1	100.0
Secondaryprogressive: No.	171	89	117	20	397
**%**	43.1	22.4	29.5	5.0	100.0
Don’t know: No.	329	132	132	32	625
**%**	52.6	21.1	21.1	5.1	100.0
	**Anxiety levels**				
**Depression levels:**					
Normal: No.	1415	498	251	23	2187
**%**	64.7	22.8	11.5	1.1	100.0
Mild: No.	316	274	265	37	892
%	35.4	30.7	29.7	4.1	100.0
Moderate: No.	143	227	382	107	859
%	16.6	26.4	44.5	12.5	100.0
Severe: No.	13	21	60	58	152
%	8.6	13.8	39.5	38.2	100.0

Anxiety and depression levels by gender and type of MS are shown, and anxiety and depression levels are compared.

### Relationships between Variables

Spearman’s rank correlation coefficient was used to assess relationships between variables. Little or no relationship was found between age and HADS score *(rho* = −0.06, *p*<0.001), or age and depression score (*rho* = 0.04, *p* = 0.10), but there was a weak negative relationship between age and anxiety score (*rho* = −0.18, *p*<0.001). Similar patterns were seen with time since first symptoms and time since diagnosis against HADS scores. That is, there was little or no relationship between time since first symptoms and HADS score (rho = −0.03, not sig), or depression score (rho = 0.045, *p* = 0.008), but there was a weak negative relationship with anxiety score (*rho* = −0.10, p<0.001). There was little or no relationship between time since diagnosis and HADS score (*rho* = −0.06, *p* = 0.01), or depression score *(rho* = 0.01, not sig), but there as a weak negative relationship between time since diagnosis and anxiety score (*rho* = −0.11, *p*<0.001); Depression and anxiety scores were positively correlated (*rho* = 0.565, *p*<0.001), similar to the reference group (0.53, *p*<0.001) [17].

As weak negative relationships were observed between the timeline variables and anxiety scores, tests were conducted to assess if there were any differences in the pattern of HADS scores between standard ONS age categories (15 to 24, 25 to 44, 45 to 64, >65 years). A significant difference was found in HADS scores between the age groups with the youngest age group having the highest mean ranks and the over 65s having the lowest mean ranks (*p*<0.001, *N* = 4096), and the results were similar for anxiety and age bands (*p*<0.001, *N* = 4294). Depression scores also differed between age bands but with a different pattern of mean ranks, the lowest being in the youngest age group and the highest being in the 45 to 64 age band (*p*<0.001, *N* = 4296).

**Figure 3:**
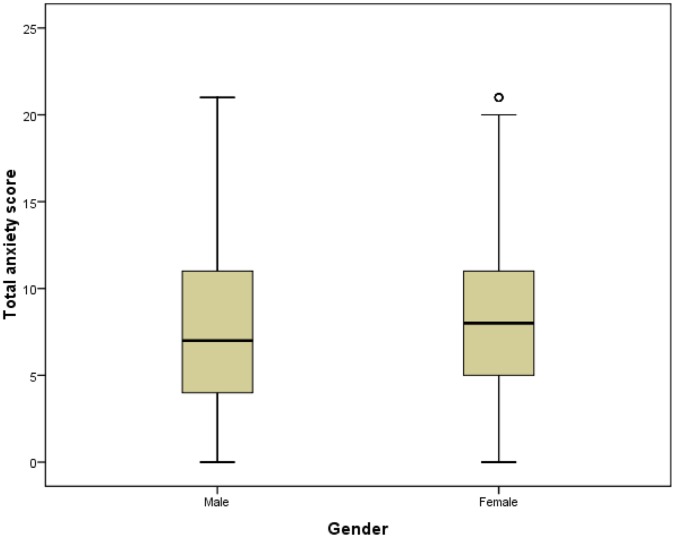
Anxiety scores for men and women. The anxiety scores for men and women are indicated. It can be seen that the women’s scores are higher than men’s.

**Figure 4:**
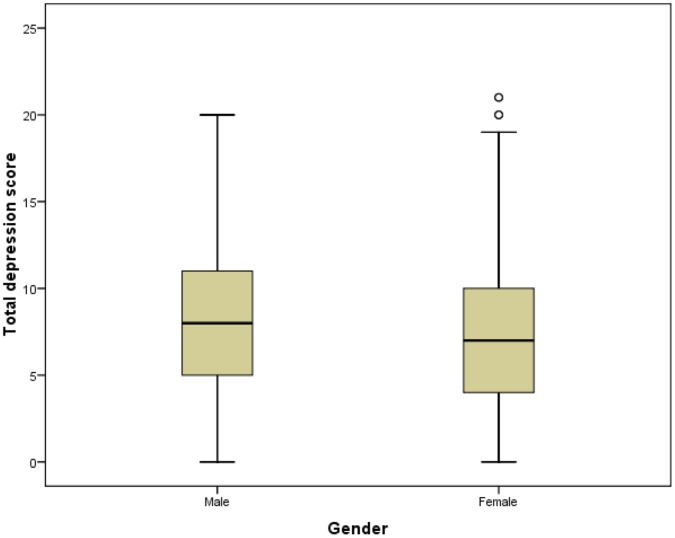
Depression scores for men and women. The depression scores for men and women are indicated. It can be seen that the men’s scores are higher than women’s.

**Figure 5:**
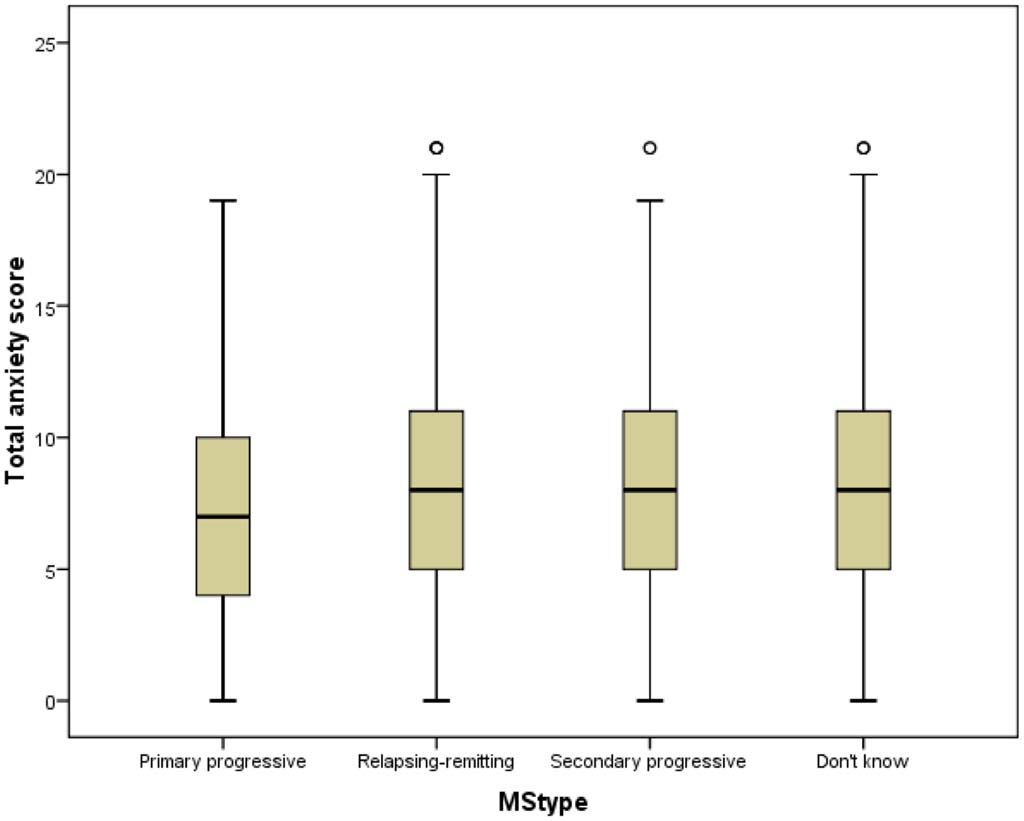
Anxiety scores by MS type. The variations in anxiety scores by type of MS are shown.

**Figure 6:**
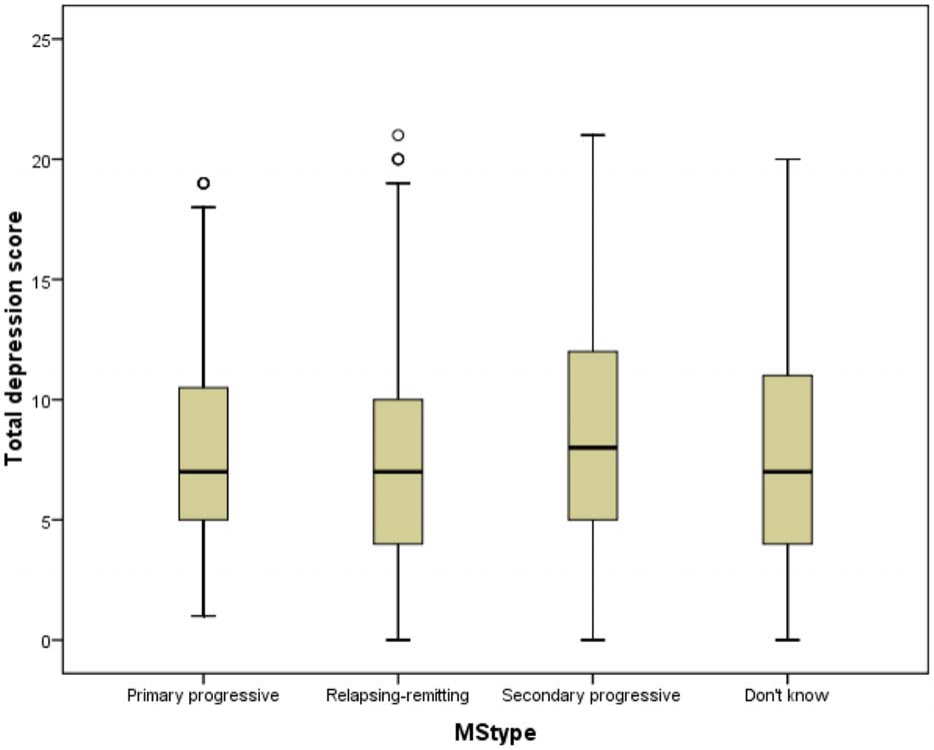
Depression scores by MS type. The variations in depression scores by type of MS are shown.

**Figure 7:**
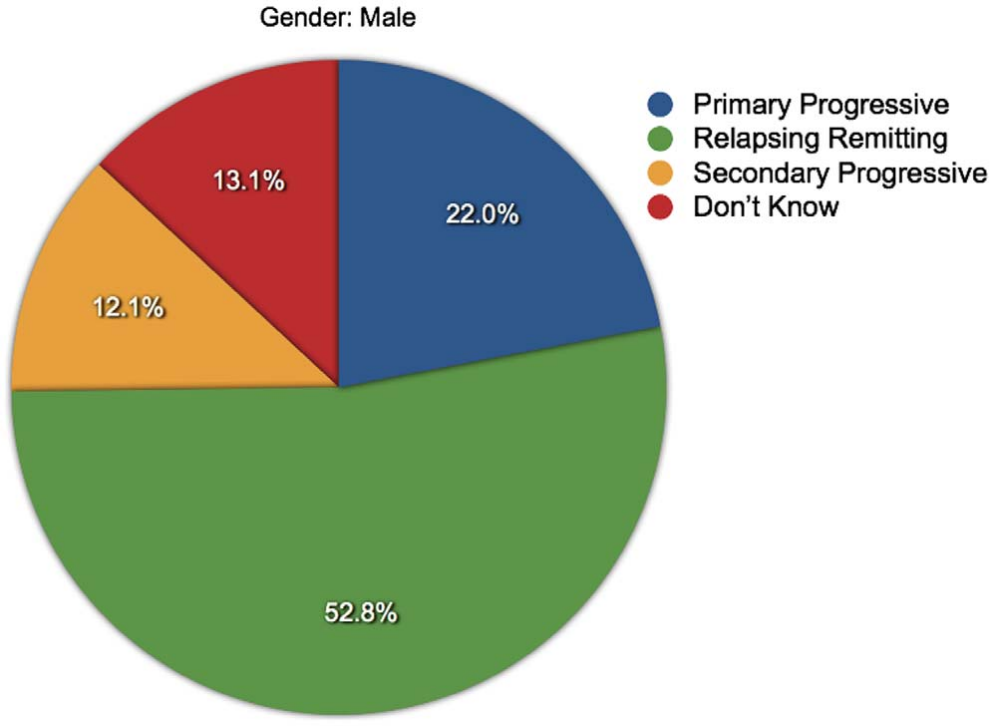
Proportions of MS types among men. The various proportions of MS types reported by the male respondents are shown.

**Figure 8:**
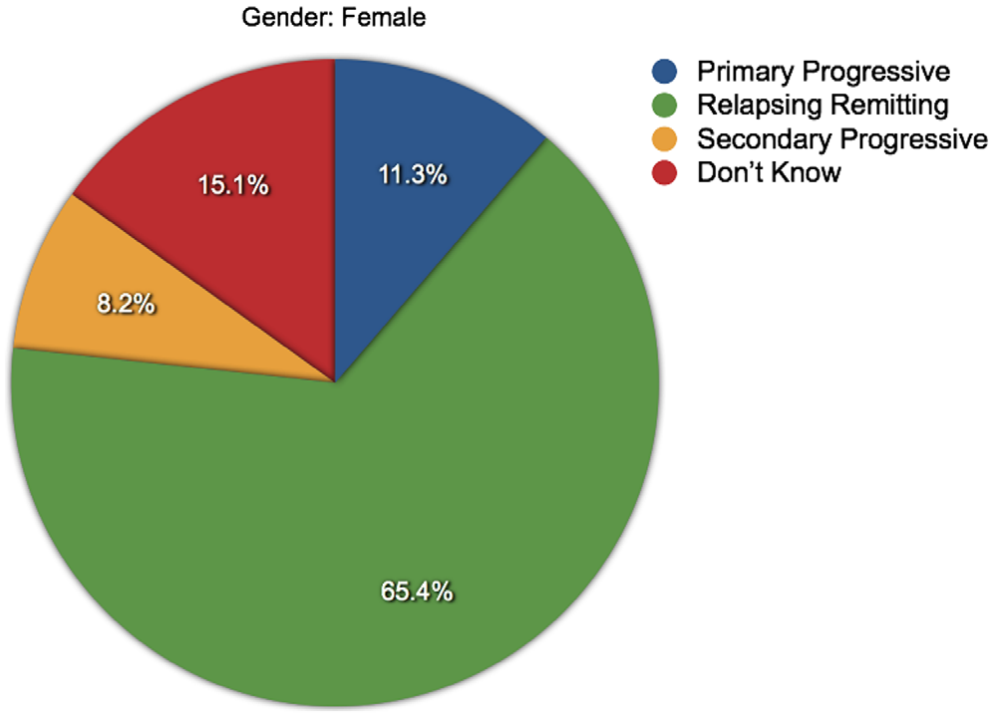
Proportions of MS types among women. The various proportions of MS types reported by the female respondents are shown.

### Frequencies of Anxiety and Depression

Over half of the respondents (54.1%) scored ≥8 for anxiety and 46.9% scored ≥8 for depression. The anxiety and depression scores were separately grouped into the accepted categories of normal (< = 7), mild (8–11), moderate (12–15) and severe (>15) [18]. These were use to explore the proportional relationships between anxiety and depression categories against MS type and gender. Men were more frequently depressed than women: 50.6% compared to 45.3%, but women were more frequently anxious than men: 56.4% compared to 48.0%. Anxiety was most frequent among people with RRMS (56.5%), whereas depression was most frequent among people with SPMS (56.9%). Inter-relationships between anxiety and depression categories were also compared: of the people who were suffering severe anxiety, over half were also experiencing moderate or severe depression (57.7%); and of the people who were reporting severe depression, over three-quarters were also suffering moderate or severe anxiety (77.7%). The proportions of people suffering from anxiety and depression are shown in Figures 1 and 2, and further details are shown in Table 2.

### Differences in Anxiety and Depression Levels

Mann-Whitney U tests were used to determine if HADS scores differed significantly between genders, but no significant difference was found (not sig, *N* = 4090). However, anxiety scores were higher in women (*p*<0.001, *N* = 4287) and depression scores were higher in men (*p*<0.001, *N* = 4290) (Figures 3 and 4). Kruskall-Wallis tests were used to assess if HADS scores varied by MS type. These showed that HADS scores, anxiety scores and depression scores all varied significantly with MS type, and with differing patterns in mean ranks: HADS (*p*<0.03, *N* = 4043), anxiety (*p*<0.001, *N* = 4233) and depression (*p*<0.001, *N* = 4235). (Figures 5 and 6). It was also noted that the types of MS were not independent of gender. For men the proportions among the different types of MS were: 22.0% PPMS, 52.8% RRMS, 12.1% SPMS and 13.1% DKMS; for women they were: 11.3% PPMS, 65.3% RRMS, 8.2% SPMS and 15.1% DKMS (chi squared *p*<0.001) (Figures 7 and 8).

These results showed that women are more anxious than men, and that anxiety is higher in people with RRMS, but greater proportions of women than men have RRMS. Conversely, men are more depressed than women, and depression is higher in people with PPMS and SPMS, but greater proportions of men than women have PPMS or SPMS. This indicates there may be some confounding between the variables under test. Additional tests were conducted to control for this possibility. Firstly Mann-Whitney U tests were used to compare anxiety and depression scores (separately) between men and women in each of the MS types. Then, Kruskall-Wallis tests were used to compare anxiety and depression scores (separately) between types of MS for each gender.

Mann Whitney U tests showed that for each of PPMS and RRMS, women were significantly more anxious than men (*p* = 0.03, *N* = 606 and *p*<0.001, *N* = 2609 respectively). Significant difference was retained for RRMS when a Bonferroni correction was applied (for a required *p* value of ≤0.0125), but not for PPMS. There was no significant difference in anxiety scores between the genders for SPMS (not sig, *N* = 396 ) or DKMS (not sig, *N* = 613). Men with RRMS or DKMS were significantly more depressed than women (*p* = 0.035, *N* = 2596 and *p* = 0.008, *N* = 625, respectively), but this was not retained when the Bonferroni correction was applied. There was no significant difference in depression scores between the genders for PPMS and SPMS (not sig, *N* = 611 and not sig, *N* = 395, respectively). Kruskall-Wallis tests showed that there was no significant difference in men’s anxiety scores between different types of MS (not sig, *N* = 1226), but women’s anxiety scores differed with type of MS (*p* = 0.017, *N* = 2998) with the highest levels in RRMS. Depression scores varied with MS type for men and for women (*p*<0.001, *N* = 1238 and *p*<0.001, *N* = 2989, respectively), with the highest levels in SPMS in men and women.

## Discussion

### Main Findings

This work used >4000 responses to the HADS questionnaire gained via the web portal of the UK MS Register to study the anxiety and depression profiles of people with MS, and to explore the relationships between anxiety and depression and various factors that might have an impact on the rates and levels of these conditions. Within the sample there were a broad range of ages and MS durations and our respondents were reasonably representative of the characteristics of prevalent MS cohorts reported elsewhere [19]–[22]. A literature review has shown that the optimal threshold for indicating likely caseness is ≥8 for each of anxiety and depression [23]; over half the respondents in this study scored ≥8 for anxiety and almost half scored ≥8 for depression. This indicates that, in common with other studies that have compared anxiety and depression rates in people with MS with those of their respective general populations [2], [5], [24], the prevalence rates observed here are higher than in the general UK population, and the mean anxiety and depression scores were higher than those found for the general UK population [17]. Furthermore, the rates are as high (or higher) as reported by earlier studies of people with MS [1], [2], [5], [24], [25].

As well as differences in frequencies of anxiety and depression, there were differences in the levels of anxiety and depression between genders and MS types. Women suffered higher levels of anxiety than men, and men were more depressed than women. The levels of anxiety and depression also varied between types of MS, with anxiety higher in people with RRMS and depression higher in people with PPMS or SPMS. However, these findings might be subject to confounding because there were different distributions of MS types between the genders. Having applied measures to control for this possibility, it was found that women with RRMS were more anxious than men with this type, and more anxious women with other types of MS. Conversely, there were no significant differences in men’s anxiety levels across the MS types. For each gender, men and women with SPMS were more depressed than men or women with other types of MS, and there were no significant differences in the depression levels of men and women with PPMS or SPMS.

There was a positive correlation between anxiety scores and depression scores indicating that the two conditions are related, and this was also seen in the general population reference group [17]. Apart from this, the majority of the analyses of linear relationships, such as age or disease duration against HADS scores showed little correlation, indicating that the prevalences of anxiety and depression are generally high across these variables. However, there were some weak negative relationships between anxiety and the three timeline variables tested. There were also some variations in anxiety and depression levels among standard age groups, with higher levels of anxiety in the 15 to 24 age group, and lower levels in the over 65 age group, and higher levels of depression in the 45 to 64 age band. It is likely that these findings are partly due to population norms, such as the greater prevalence of anxiety among women compared to men, and varying levels of anxiety and depression associated with population maturation. But it is also possible that the findings are partly due to the patterns of initial diagnosis and disease course. The majority of people are initially diagnosed with RRMS (up to 85%) and fewer with PPMS (up to 15%). Over time there can be a sustained build-up of disability, independent of relapses, so that in about two-thirds of people with RRMS, the diagnosis may change to SPMS [22].

### What this Study Adds

This is the largest known study of this type among people with MS to be reported on to date. Its novel mode of data collection has generated a large sample size which has allowed more in-depth, robust sub-group analysis to be conducted. It has brought new information on anxiety and depression from the respondents’ own perspectives. It has shown that anxiety and depression are highly prevalent in people with MS and that the rates and levels differ with various factors. It has provided greater knowledge of the anxiety and depression profiles of people with MS that can be used for care planning and research.

### Limitations

This study was based on self-reported information and the respondents were not necessarily a fully representative sample of people with MS in the UK. Ascertainment is commonly assumed to be skewed when using web-based data collection methods, as the technology may pose a barrier to the elderly, disadvantaged, technically inexperienced or cognitively impaired [26], [27]. It is also possible that help-seeking behavior among people experiencing anxiety and depression is leading to a greater apparent prevalence of these conditions. Any response bias in portal data will be addressable using the linkable data from clinical sites and routine sources, as the Register continues to build up an increasingly rich picture of MS in the UK.

### Future Work

Future work will analyse the responses to other questionnaires delivered via the platform, to study impact on daily life, influence of lifestyle factors, medication records and health outcomes for people with MS. We will be able to study the responses to the HADS questionnaire in association with self-reported medication records to explore the relationships between anxiety and depression and medicines in use. Data for the Register continues to be collected, and we will link self-reported information with clinical and administrative Register data to compare information between the data sources. For example, we aim to use the Register make a more in-depth study of anxiety in people with MS, since it has been noted that this is under-studied compared to depression [1]. We will also be able to study the relationships between disability and mental well-being. As well as this, a programme of qualitative research is underway to engage with people with MS to improve our understanding of their needs in contributing to the Register [28].

### Conclusion

The notably high rates and levels of anxiety and depression among people with MS found in this study indicate that their mental health needs could be better addressed. The importance of appropriate mental health treatment and care and their potential to improve quality of life for people with MS has been highlighted [29], [30]. The disease course of MS can be highly unpredictable and each case must be managed individually, so it is important for service planners to be reflective of the potential additional demand for mental health services as well as other medical and rehabilitative care. The findings of this study may be used to support service planning and further research to provide the best outcomes for people with MS to help alleviate these debilitating conditions.

## Supporting Information

Table S1 **Supplementary information on mean ranks from Kruskall-Wallis and Mann-Whitney U tests.** The mean rank scores for the Kruskall-Wallis and Mann-Whitney U tests reported in the text are shown.
(DOCX)
