# Supplementary material for: A Large-Scale Study of Anxiety and Depression in People with Multiple Sclerosis: A Survey via the Web Portal of the UK MS Register
Source: PLoS One. 2012 Jul 30;7(7):e41910. doi: 10.1371/journal.pone.0041910 (PMC3408498; doi:10.1371/journal.pone.0041910)
Supplement: Table S1 — Supplementary information on mean ranks from Kruskall-Wallis and Mann-Whitney U tests. The mean rank scores for the Kruskall-Wallis and Mann-Whitney U tests reported in the text are shown. (DOCX) [file pone.0041910.s001.docx]

| **Test** | **For difference in scores** | **Mean ranks** |
| --- | --- | --- |
| Kruskall-Wallis | HADS between ONS age bands, 15 to 24, 25 to 44, 45 to 64, >65 years | 2207: 2080: 2099: 1745 |
| Kruskall-Wallis | Anxiety between ONS age bands, 15 to 24, 25 to 44, 45 to 64, >65 years | 2780: 2356: 2132: 1706 |
| Kruskall-Wallis | Depression between ONS age bands, 15 to 24, 25 to 44, 45 to 64, >65 years | 1884: 1992: 2259: 2028 |
| Mann-Whitney U | HADS between genders | M 2040: F 2048 |
| Mann-Whitney U | Anxiety between genders | M 1982: F 2210 |
| Mann-Whitney U | Depression between genders | M 2282: F 2089 |
| Kruskall-Wallis | HADS between MS types | 1944 PPMS: 2009 RRMS: 2191 SPMS: 1,997 DKMS |
| Kruskall-Wallis | Anxiety between MS types | 1920 PPMS: 2172 RRMS: 2090 SPMS: 2093 DKMS. |
| Kruskall-Wallis | Depression between MS types | 2282 PPMS: 2031 RRMS: 2434 SPMS: 2116 DKMS |
| Mann-Whitney U | Anxiety between genders for PPMS | M 286: F 317 |
| Mann-Whitney U | Anxiety between genders for RRMS | M 1206: F 1338 |
| Mann-Whitney U | Anxiety between genders for SPMS | M 189: F 204 |
| Mann-Whitney U | Anxiety between genders for DKMS | M 287: F 314 |
| Mann-Whitney U | Depression between genders for PPMS | M 318: F 296 |
| Mann-Whitney U | Depression between genders for RRMS | M 1352: F1281 |
| Mann-Whitney U | Depression between genders for SPMS | M 210: F 191 |
| Mann-Whitney U | Depression between genders for DKMS | M 345: F302, |
| Kruskall-Wallis | Anxiety between types of MS in men | PPMS 570: RRMS 630: SPMS 622: DKMS 611 |
| Kruskall-Wallis | Anxiety between types of MS in women | PPMS 1,372: RRMS 1,530: SPMS 1,480: DKMS 1,475 |
| Kruskall-Wallis | Depression between types of MS in men | PPMS 647: RRMS 581: SPMS 709: DKMS 646 |
| Kruskall-Wallis | Depression between types of MS in women | PPMS 1616: RRMS 1,451: SPMS 1,710: DKMS 1,477 |
